# Human γδ T cell Recognition of lipid A is predominately presented by CD1b or CD1c on dendritic cells

**DOI:** 10.1186/1745-6150-4-47

**Published:** 2009-12-01

**Authors:** Yongchun Cui, Lei Kang, Lianxian Cui, Wei He

**Affiliations:** 1 Department of Immunology, Institute of Basic Medical Sciences, Chinese Academy of Medical Sciences & School of Basic Medicine, Peking Union Medical College, 5 Dong Dan San Tiao, Beijing 100005, China

## Abstract

**Background:**

The γδ T cells serve as early immune defense against certain encountered microbes. Only a few γδ T cell-recognized ligands from microbial antigens have been identified so far and the mechanisms by which γδ T cells recognize these ligands remain unknown. Here we explored the mechanism of interaction of human γδ T cells in peripheral blood with Lipid A (LA).

**Results:**

First, resting γδ T cells (mainly Vδ2 T cells) displayed a strong proliferative response to LA-pulsed monocyte-derived dendritic cells (moDC) and LA-pulsed paraformaldehyde-fixed moDC, but not to free LA in a TCR γδ-dependent manner. Second, anti-CD1b or anti-CD1c antibodies could block proliferative response of resting γδ T cells to LA-loaded moDC. Besides, only LA-loaded CD1b/CD1c-transfected C1R lymphoblastoma cells (CD1b-/CD1c-C1R) were able to stimulate the proliferation of human γδ T cells. Third, the expressions of both Toll-like receptor (TLR)2 and TLR4 on surface of LA-activated γδ T cells were upregulated, whereas only anti-TLR4 antibody could partially block their response to LA; Finally LA-loaded moDCs induce γδ T cells to produce Th1 cytokines, such as IFN-γ.

**Conclusion:**

Taken together, we found a novel mechanism that human γδ T cells recognize LA in a CD1b- or CD1c-restricted manner in first response against Gram-bacteria, while the interaction between TLR4 on γδ T cells and LA might strengthen the subsequent response of γδ T cells.

**Reviewers:**

This article was reviewed by Hao Shen, Youwen He (nominated by Dr. Laurence C Eisenlohr), Dr. Michael Lenardo and Dr. Pushpa Pandiyan.

## Background

Although T cells bearing the TCRγδ represent a minor subset of T cells in the periphery, they are abundant in epithelial and mucosal tissues, the sites of initial host invasion by many pathogens. Many studies demonstrated that TCRγδ^+ ^T cells could have a sentinel role in the early host response against parasitic [1], bacterial [2] and viral infections [3]. The γδ T cells can recognize protein antigens in a MHC-unrestricted manner. Moreover, γδ T cells can also recognize non-peptide molecules, such as microbial metabolites (pyrophosphomonoesters and alkyl amines) [4,5] and synthetic aminobisphosphonates (pamidronate) [6] directly via TCRγδ. In addition, several studies demonstrated that the interaction of T cells with lipid/glycolipid antigens is associated with MHC-like cluster of differentiation (CD) molecules [7,8], however, most such experiments have focused on αβ T cells or γδ T cells in mice. The mechanism of human γδ T cells recognizing lipid antigens is largely unknown.

Lipid A (LA) is the most conservative membrane anchor of LPS and is regarded to be responsible for LPS-induced biological effects [9,10]. The basic structure of LA consists of a β-1, 6-linked glucosamine disaccharide substituted with two negatively charged phosphates and saturated fatty acids. It has strong antigenicity. So we select LA as antigen to further understand the potential biological effect of human γδ T cells in the immune response against invasive bacteria. Here, we want to know whether antigen presenting cells (APCs), such as monocyte-derived dendritic cells (moDC), are able to present LA via CD1 family members to resting γδ T cells (mainly Vδ2 T cells) from peripheral blood. Furthermore, it has been demonstrated that murine γδ T cells could directly interact with LA via Toll like receptor (TLR) 2/4 on their surface [11,12], so we try to explore the TLR2/4 expression on the surface of human γδ T cells when activated by LA-pulsed moDC.

In present study, we have attempted to address three questions. First, can LA induce human γδ T cells to proliferate in a CD1-restricted manner? Second, which CD1 family member is responsible for LA-induced activation of the γδ T cells? Third, besides TCR, whether LA in the context of CD1 complex activates human γδ T cells via other receptors, such as TLR2/4?

## Results

### LA-induced proliferation of γδ T cells requires moDC

**Figure 1:**
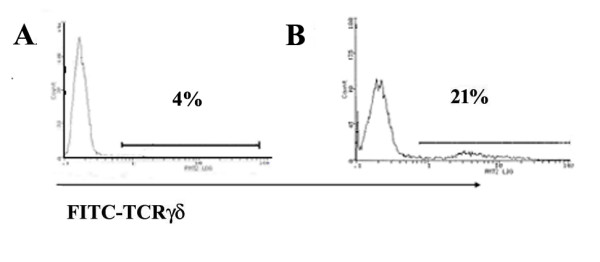
**The percentage of γδ T cell in freshly isolated PBMCs and γδ T cell-enriched PBMCs**. The data were obtained by flow cytometry using FITC-conjugated anti-TCRγδ mAb. Similar results were observed in other five healthy donors.

**Figure 2:**
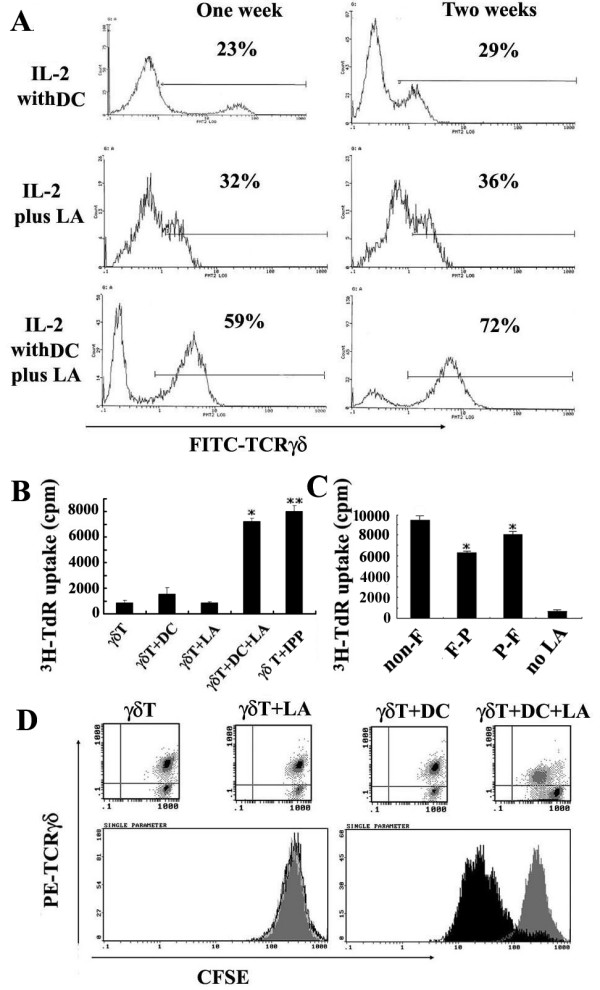
**Proliferative response of human γδ T cells to LA in the presence of moDC**. (A) The percentage of γδ T cells was determined by flow cytometry. Similar results were observed in other five healthy donors. (B) In ^3^H-TdR incorporation assay, purified γδ T cells (99% purified) were co-cultured with LA in the presence or absence of moDC. Results are shown as cpm (mean ± SD) of five experiments. * *P *< 0.05 vs. γδ T, γδ T+LA or γδ T+DC respectively, * *P < 0.05 vs. γδ T. (C) Irradiated moDCs were fixed before or after LA stimulation. The proliferation of γδ T cells was determined as described in *Methods and Materials*. *P > 0.05 vs nonfixed moDCs. (D) The reduction of fluorescence of CFSE labeled γδ T cell-enriched PBMCs was detected after 72 h by gating on TCRγδ^+ ^population. The top row shows dot plots; the bottom row shows the overlaps of histograms corresponding to top dot plots, among which the left histograms are the overlaps of γδT (black line histogram), γδ T+LA (dark grey filled histogram) and γδ T+DC (light grey filled histogram), and the right histograms are the overlap of γδ T+LA (dark grey filled histogram) and γδ T+LA+DC (black filled histogram). All experiments were performed at least three times with consistent results. γδ T: γδ T cells; LA: lipid A; DC: monocyte derived dendritic cells (moDC), IPP: isopentenyl pyrophosphate. These abbreviations above also appear in Fig. 2, 3, 4 and 5. Non-F, P-F or F-P respectively represent the groups, in which non-fixed moDCs, moDCs pulsed with LA before fixation, or moDCs fixed before pulsed with LA were used (Fig. 2C).

**Figure 3:**
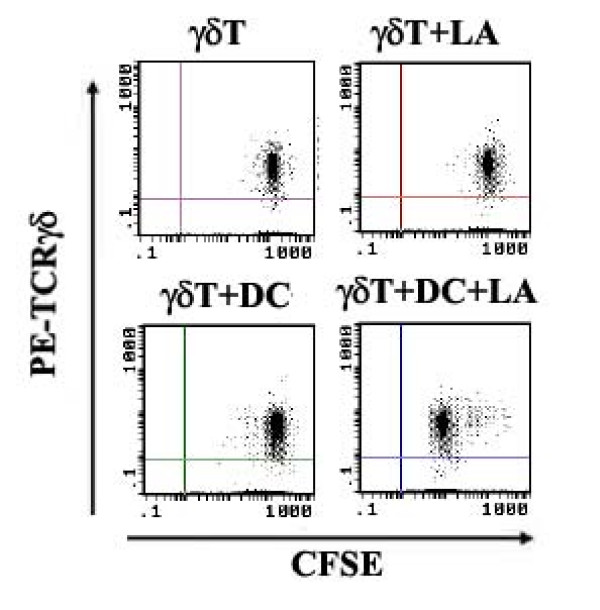
**Proliferative response of purified human γδ T cells to LA in the presence of moDC in CFSE dilution assay**. CFSE labeled purified human γδ T cells were co-cultured with LA in the presence or absence of irradiated autologous moDC, plus IL-2 (20 U/ml). The reduction of CFSE fluorescence was detected after incubation for 72 h by gating on TCRγδ^+ ^population. All experiments were performed at least three times with consistent results.

To investigate LA-induced activation of γδ T cells, γδ T cell-enriched PBMCs from healthy donors were incubated for one or two weeks with LA, with or without irradiated autologous moDC plus low doses of exogenous IL-2 (10 U/ml). Depleting TCRαβ^+ ^and CD4^+ ^T cells in PBMC increased the proportion of γδ T cells in PBMC from 4% (Fig. 1A) to 21% (Fig. 1B) in one representative donor. However, stimulation with LA in the presence of moDC and IL-2 increased the percentage of γδ T cells dramatically (59% at week 1 and 72% at week 2) (Fig. 2A). In contrast, IL-2 with or without LA induced a modest expansion of γδ T cells, when moDCs were excluded in the culture (Fig. 2A), suggesting that LA can induce significant expansion of γδ T cells in the presence of autologous moDC. As shown in Fig. 2B, proliferations of γδ T cells in these different groups as assessed by ^3^H-TdR incorporation assay was consistent with that seen in flow cytometry analysis. Moreover, in CFSE dilution assay, no matter using γδ T cell-enriched PBMC (Fig. 2D) or purified γδ T cell (Fig. 3), γδ T cells proliferated significantly after stimulated with LA plus IL-2 in the presence of moDCs. These results strongly suggest that LA-pulsed moDC induced human γδ T cell proliferation.

### LA-loaded moDCs induce the proliferation of human γδ T cells

The action of moDCs during LA interaction with human γδ T cells was assessed by means of pulse-fixation experiments. In these experiments, moDCs were pulsed with LA (10 ng/ml) before or after fixation with paraformaldehyde. Cells were extensively washed after LA pulse and therefore there was no free (soluble) antigen present at the time of γδ T-cell addition. As shown in Fig. 2C, the LA-pulsed moDCs before and after fixation were both able to activate γδ T cells and there is no significant difference comparing those without fixation (*P > 0.05 vs nonfixed moDCs). This experiment suggests that LA do stimulate human resting γδ T cells to proliferate by loading on moDCs.

### LA recognition by human resting γδ T cells depends on CD1b and CD1c

**Figure 4:**
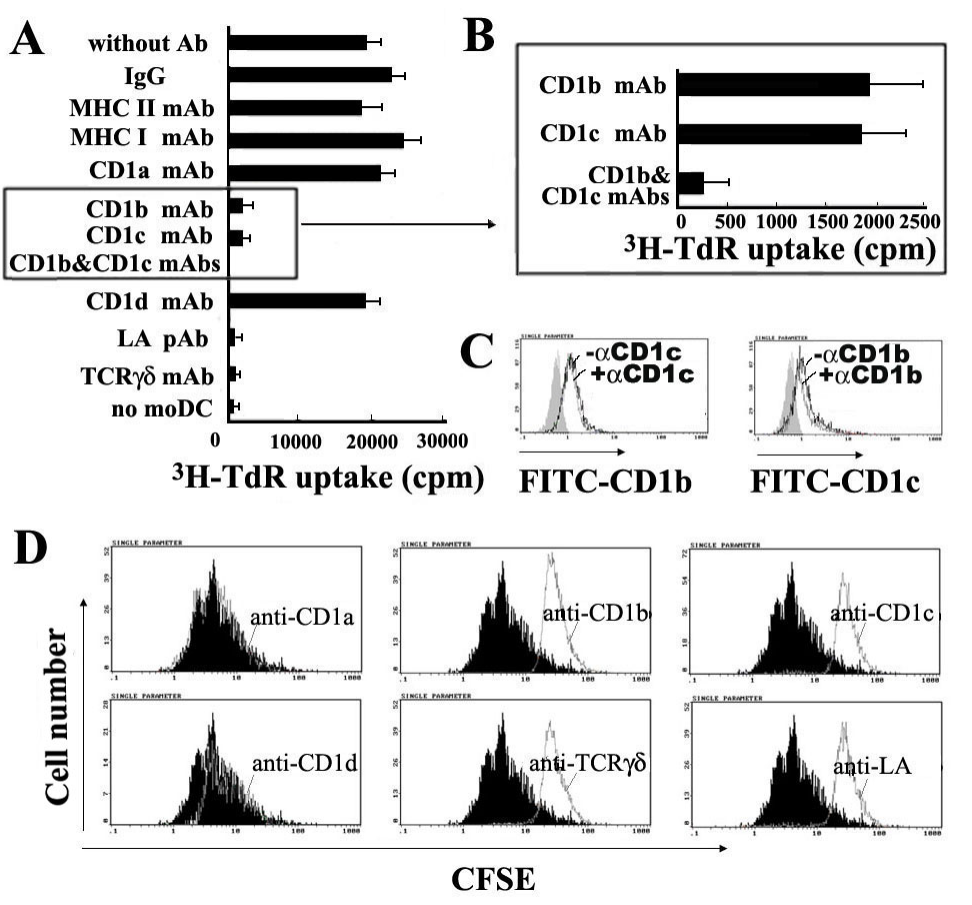
**Antibody blocking assay confirmed that both CD1b and CD1c molecules on moDC could be important for LA-induced proliferations of γδ T cells**. In the group of γδ T cell+LA+DC+anti-TCRγδ mAb, γδ T cells were preincubated with anti-TCRγδ mAb at 37°C for 2 h; in the group of γδ T cell+LA+DC+anti-LA, LA-loaded moDCs were preincubated with pAb anti-LA for another 2 h; In other groups, autologous irradiated moDCs were preincubated with mAbs against MHC I, MHC II, CD1a, CD1b, CD1c, CD1d, or CD1b plus CD1c respectively. Then, they were thoroughly washed. (A and B). The proliferations of γδ T cells were determined by ^3^H-TdR incorporation. The results are expressed as mean ± SD of three independent experiments. (C) The moDCs were incubated with or without purified anti-CD1b or CD1c mAb for 2 h, then stained the CD1b-blocked moDC with FITC-conjugated anti-CD1c mAb and stained the CD1c-blocked moDC with FITC-conjugated anti-CD1b mAb, analyzed CD1b^+ ^and CD1c^+^moDCs by flow cytometry. (D) CFSE labeled γδ T cell-enriched PBMCs were co-cultured with moDC and LA, with or without purified Abs against CD1a, CD1b, CD1c, CD1d, TCRγδ and LA. The reduction of CFSE fluorescence was detected after 72 h by gating on TCRγδ^+ ^populations. Histograms show overlaps of the group of γδ T cell+LA+moDC without any antibody (black filled histograms) and the groups with anti-CD1a, -CD1b, -CD1c, -CD1d, -TCRγδ or -LA antibody (grey line histograms), respectively. All experiments were performed at least three times with consistent results. Note: -αCD1b/CD1c: without anti-CD1b/CD1c mAbs; +α CD1b/CD1c: with anti-CD1b/CD1c mAbs.

**Figure 5:**
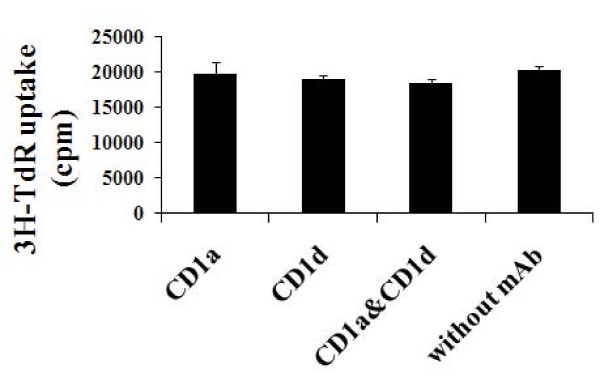
**LA recognition by human resting γδ T cells independent of CD1a and CD1d**. In the groups of purified γδ T cell+LA+DC+anti-CD1a/CD1d or CD1a plus CD1d mAb, autologous irradiated moDCs were preincubated with mAbs against CD1a, CD1d, or CD1a plus CD1d respectively. Then, they were thoroughly washed. The proliferations of γδ T cells were determined by ^3^H-TdR incorporation. The results are expressed as mean ± SD. *P > 0.05 vs the group with anti-CD1d mAb alone, anti-CD1a alone or control group (without mAb) respectively.

**Figure 6:**
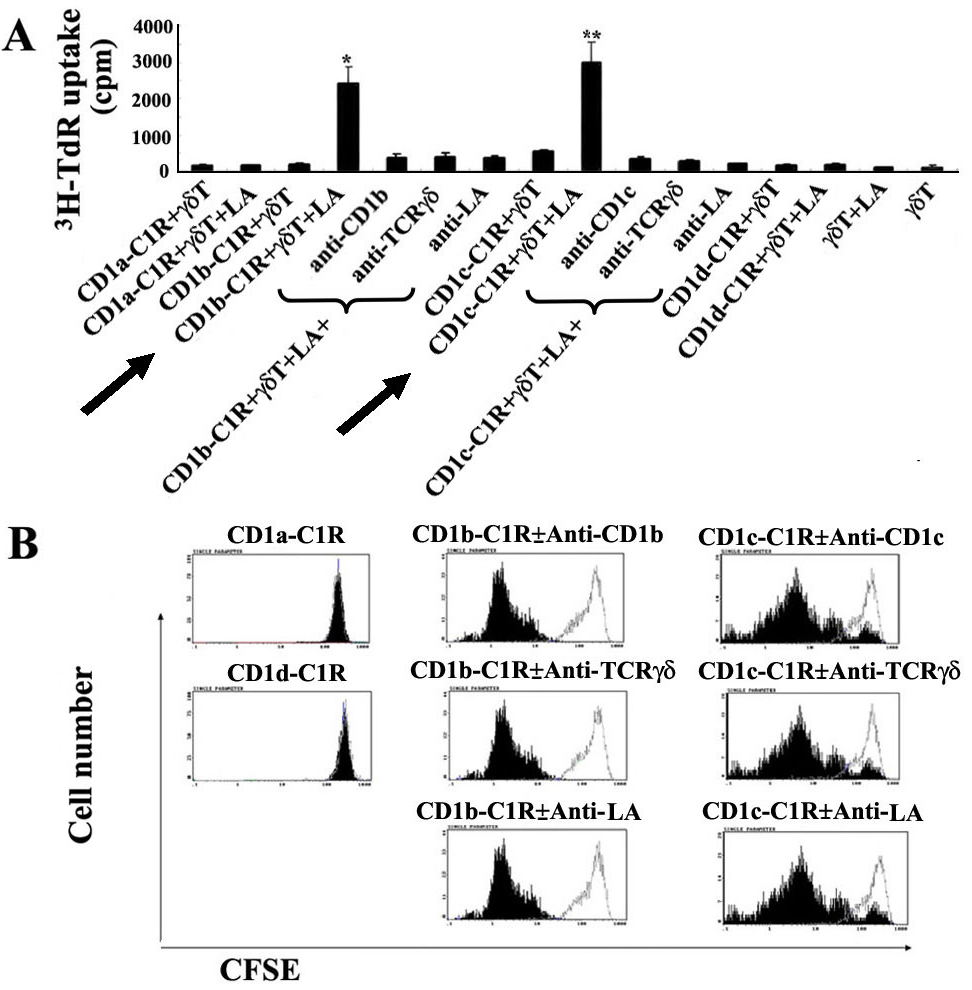
**Human γδ T cells responded to LA-loaded CD1b/CD1c-transfected C1R cells, but not to LA-loaded CD1a/CD1d-transfected C1R cells**. Purified γδ T cells and γδ T-enriched cells were prepared as described in *Materials and Methods*. CD1-transfected C1Rs are irradiated (4,000 rads) before use and preincubated with LA for the groups plus LA. (A) ^3^H-TdR-incorporation assay using purified human γδ T cells. *P < 0.05, when compared the arrow-marked group (CD1b-C1R+γδ T cell+LA) with those including CD1b-C1R+ γδ T cell, CD1b-C1R+γδ T cell+LA+anti-CD1b mAb, CD1b-C1R+γδ T cell+LA+anti-TCRγδ mAb, or CD1b-C1R+ γδ T cell+LA+anti-LA pAb; **P < 0.05 when compared the arrow-marked group (CD1c-C1R+ γδ T cell+LA) with those including CD1c-C1R+ γδ T cell, CD1c-C1R+ γδ T cell+LA+anti-CD1c mAb, CD1c-C1R+γδ T cell+LA+anti-TCRγδ mAb or CD1c-C1R+ γδ T cell+LA+anti-LA pAb. (B) CFSE labeled γδ T cell-enriched PBMCs were co-cultured with CD1a-, CD1b-, CD1c- and CD1d-transfected C1R cells preincubated with LA, with or without purified blocking Abs. The reduction of CFSE fluorescence was detected after 72 h by gating on TCRγδ^+ ^populations. Histogram overlaps indicate the group of γδ T cell+LA+CD1-transfected C1Rs without any antibody (black filled histograms) and the groups with anti-CD1a/-CD1d (left panel), -CD1b (middle panel), -CD1c (right panel), -TCRγδ or -LA (middle & right panels) antibodies (grey line histograms), respectively. All experiments were performed at least three times with comparable results. CD1a/b/c/d-C1R: CD1a/b/c/d-transfected C1R cell lines.

To identify the antigen-presenting molecules on APC that present LA to γδ T cells, we performed antibody blocking assay. As shown in Fig. 4A, B and 4D, the mAbs against CD1b, CD1c, or TCRγδ and pAb against LA could completely block the proliferative response of human γδ T cells to LA, while mAbs against CD1a, CD1d (alone or combined with CD1a, see Table 1 and Fig 5), MHC I or MHC II did not have such a blocking effect, suggesting that LA-loaded moDCs induce human γδ T cell to proliferate in a CD1b-/CD1c-restricted and TCRγδ-dependent manner. The degrees of blockages for anti-CD1b and CD1c mAbs are identical (Fig. 2B). To further confirm the above observations, we use CD1a-, CD1b-, CD1c- and CD1d-transfected C1R cells as APCs to stimulate purified human γδ T cells after loaded by LA. Similar results were observed in ^3^H-TdR incorporation assay (Fig. 6A) and CFSE dilution assay (Fig. 6B). LA-loaded CD1b- or CD1c-C1R cells were able to significantly induce the proliferation of human γδ T cell, which can be blocked by anti-CD1b or -CD1c, anti-TCR γδ mAbs and anti-LA pAb. To determine whether antibodies against CD1b and CD1c cross-block each other, we used FITC-conjugated mouse anti-pan-CD1c or -CD1b to label moDCs which were preincubated with purified mAbs against CD1b or CD1c. As shown in Fig. 4C, CD1b^+ ^moDCs accounted for 84% (black line histogram) and 83% (grey line histogram) of the cells, respectively, before and after the blockade by anti-CD1c mAb; CD1c^+ ^moDCs accounted for 72% (black line histogram) and 64% (grey line histogram) before and after the blockade of anti-CD1b mAb, indicating that the purified anti-CD1b and -CD1c mAbs do not cross-block each other.

**Table 1:** LA recognition by human resting γδT cells independent of CD1a and CD1d

Groups	cpm			Mean	SD
CD1a mAb	19340	21635	18098	19691	1794.4
CD1d mAb	19674	18765	18886	19108.3	493.6
CD1a&CD1d mAbs*	18702	18920	17665	18429	670.6
without mAb	20065	19876	20992	20311	597.3

**Key**. The proliferation of purified human γδT cells were determined by ^3^H-TdR incorporation assay after incubating with preblocked LA-moDC by CD1d mAb alone or combined with CD1a. The group without any mAb was used as control. *P > 0.05 vs the group with CD1d mAb alone, CD1a alone or control group (without mAb).

### V δ2^+ ^cell-predominant LA-reactive γδ T cells produced Th1, not Th2 cytokines

**Figure 7:**
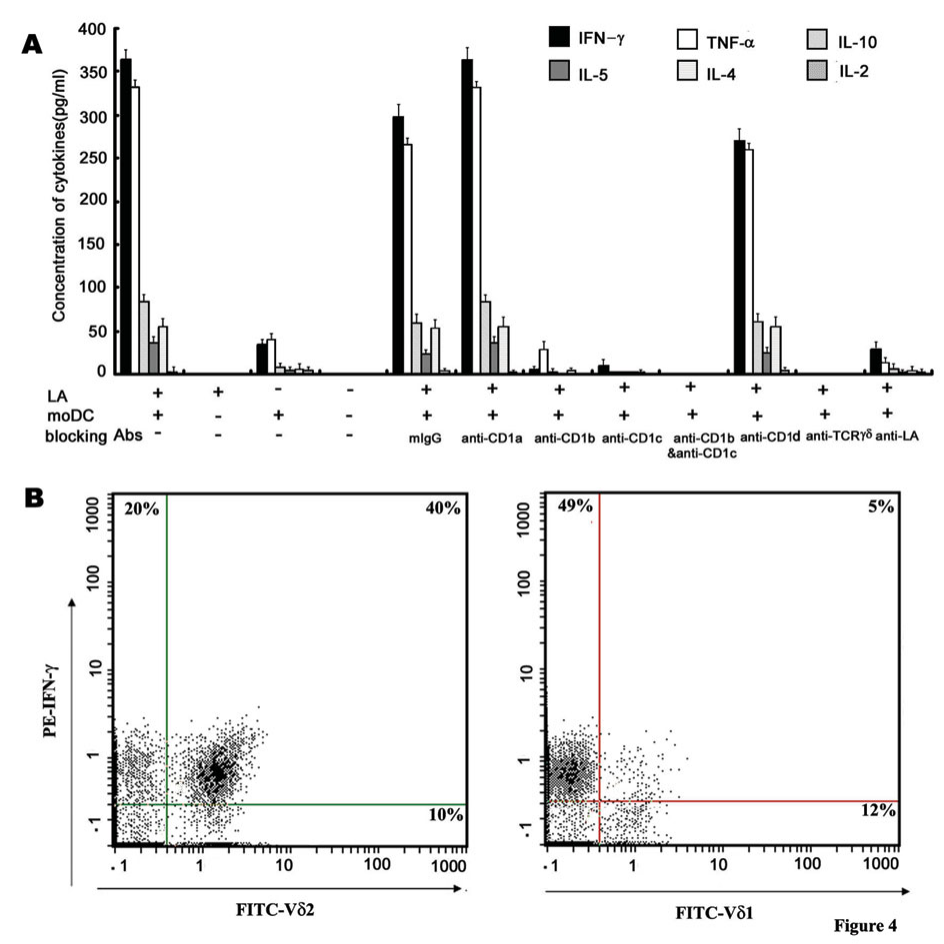
**LA-loaded moDC induced Th1 cytokine production of γδ T cells, predominantly TCRVδ2^+ ^T cells**. (A) Purified γδ T cells were incubated for 48 h with or without LA, in the presence or absence of moDCs, plus or not plus blocking antibody. For a determination of the cytokine secretion, supernatants were collected and cytokine concentration was measured by CBA kit. The results represent mean ± SD of three independent experiments. (B) γδ T-enriched PBMCs are stimulated by LA-loaded moDC for 2 weeks, then collected and double stained as described in Materials and Methods using human FITC-TCRVδ2/PE-IFN-γ (Left), and FITC-TCRVδ 1/PE-IFN-γ (right). One of two similar experiments is shown.

Several studies have shown that γδ T-cell stimulation induces the release of a variety of cytokines, particularly T helper type 1 (Th1) cytokines (IFN-γ and TNF-α)[13]. To investigate the functional consequences of γδ T-cell stimulation by LA-loaded moDC, we measured cytokine concentrations in these cultures. As shown in Fig. 7A, LA-loaded moDC induced γδ T cells to produce higher level of Th1 than Th2 type cytokines. The cytokine level produced by γδ T cells with LA-loaded moDC was much higher than that without moDC. IFN-γ and TNF-α were detected at high level, whereas IL-10, IL-5 and IL-4 were detectable at low level (less than 100 pg/ml). Interestingly, IL-2 was hardly detected in this experimental system in vitro. Consistent with the proliferation assay, antibodies against CD1b, CD1c, TCR γδ or LA could block cytokine production by γδ T cells. After co-cultured with LA-loaded moDC for 2 weeks, LA-reactive γδ T-enriched cells were collected, and analyzed for TCRVδ 1^+ ^and TCRVδ2^+^γδ T cells and cytokine production. As shown in Fig. 7B, we found that TCRVδ2^+^γδ T cells accounted for about 50% of the total population. Among these TCRVδ2^+^γδ T cells, 80% of them generated INF-γ. In contrast, TCRVδ 1^+^γδ T cells accounted for 17% of the total cells, and the percentage of INF-γ generating TCRVδ 1^+^γδ T cells among them was below 30%. Thus, TCRVδ2^+^γδ T cells are the main subset within LA-reactive γδ T cells.

### LA loaded moDCs could induce upregulated expressions of TLR4 and TLR2 on human γδ T cells and the response of LA-activated γδ T cells to LA is associated with TLR4

**Figure 8:**
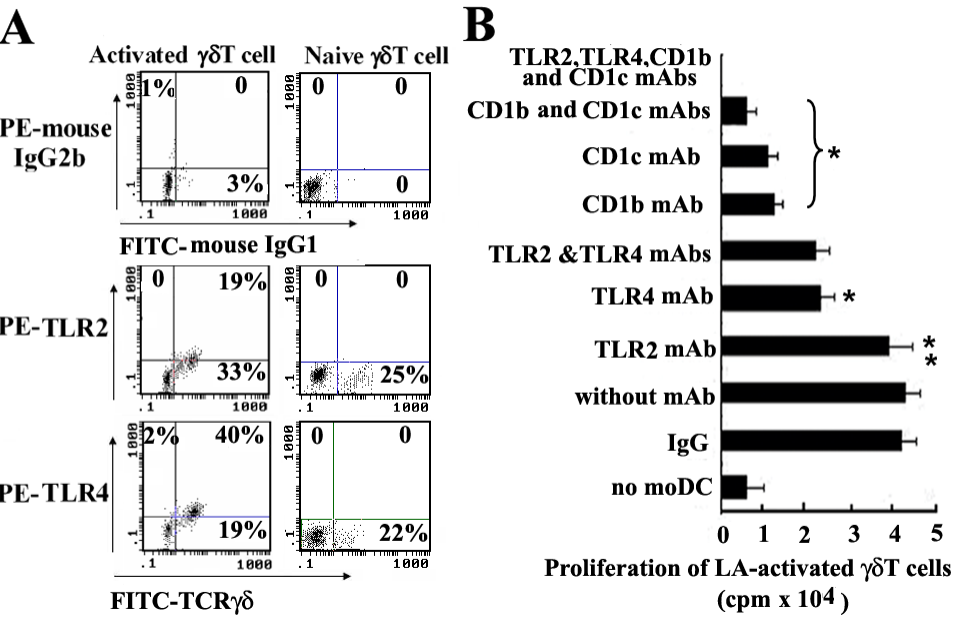
**LA loaded moDCs induce upregulated expressions of TLR4 and TLR2 on human γδ T cells and the response of LA-activated γδ T cells to LA is closely associated with TLR4**. (A) Tested cells were stained with FITC-conjugated anti-TCRγδ mAb and PE-conjugated anti-TLR4/TLR2 mAbs, and then determined by flow cytometry. The resting purified γδ T cells did not express TLR4 or TLR2 (right panel). After 7 days' co-culture with LA-loaded moDCs, TLR2^+ ^TCRγδ^+ ^and TLR4^+ ^TCRγδ^+ ^T cells accounted for 19% and 40% of tested cells, respectively (left panel). Up-panel is isotype antibody control. This result is shown as a representative experiment of three independent ones. (B) LA-activated γδ T cells were incubated with mAb TLR2, TLR4 or TLR2 plus TLR4 and rested for 24 h in medium. Autologous moDCs were preincubated with mAbs against CD1b, CD1c CD1b plus CD1c mAb at 37°C for 2 h. Then, they were thoroughly washed respectively, co-cultured with LA for 48 hour. During the last 8 h, 1 μCi of ^3^H-TdR per-well was added. ^3^H-TdR incorporation was measured. The results are expressed as mean cpm ± SD of five independent experiments. *P < 0.05, **P > 0.05, when compared with the group without mAb.

Among resting γδ T cells, we hardly found TLR4^+ ^TCRγδ^+ ^T cells or TLR2^+ ^TCRγδ^+ ^T cells. After 7-day co-culture with LA-loaded moDCs, the activated TLR2^+^TCRγδ^+ ^T cells and TLR4^+ ^TCRγδ^+ ^T cells respectively occupy 19 ± 5% and 40 ± 2% of tested cells. Fig. 8A shows the results of one representative experiment. Accordingly, in order to identify if human activated γδ T cells respond to LA via TLR pathway, we perform another proliferation assay in which LA-activated γδ T cells were rested for 24 h before preincubating with mAbs against TLR2, TLR4 or TLR2 plus TLR4. On the other hand, autologous moDC were preincubated with mAbs against CD1b, CD1c, CD1b plus CD1c mAb and then co-cultured with LA. ^3^H-TdR incorporation showed that mAbs anti-CD1b or anti-CD1c could significantly block the proliferation of LA-activated γδ T cells (P < 0.05), which became remarkable when they were applied in combination and showed a complete blockage with anti-TLR4. The mAb anti-TLR4 could partially block this proliferation (P < 0.05), while mAb anti-TLR2 did not have such blocking effect (P > 0.05) suggesting that the mechanism of human LA-activated γδ T cells response to LA involves TLR4 pathway.

## Discussion

γδ T cells play an important role in host immune defense and immunoregulation, which have been confirmed to relate to CD1 family. The γδ T cells with Vδ 1 TCR were found to able to recognize self CD1c molecules even in the absence of foreign antigens [14]. These Vδ 1 T cells proliferated in response to CD1^+ ^presenter cells, lysed CD1^+ ^targets, and released Th1 cytokines. Therefore, these cells may directly mediate host defense even before foreign antigen-specific T cells have differentiated. Moreover, these CD1-restricted Vδ 1 T cells can mediate the maturation of DCs via cell-cell contact and cytokine-related (mainly by TNF-α) manners [15]. Besides, CD1 molecules as presenting molecules have been early noted. For example, CD1 molecules are found to be necessary for αβ T cells to recognize lipid antigens [16]. In this context, a purified CD1b-restricted antigen of mycobacterium tuberculosis, mycolic acid, is presented to αβ T cells by CD1b in a TCRαβ-dependent manner. There is also data showing CD1c-mediated T-cell (mainly TCRαβ^+ ^cells) recognition of isoprenoid glycolipids in mycobacterium tuberculosis infection [14,17]. However, there is no a direct evidence showing human γδ T cells to recognize lipid antigens so far.

In present study, we demonstrate that both CD1b and CD1c molecules on moDC are required for human resting γδ T cells to recognize LA, suggesting that the interaction of human γδ T cell with lipid A is similar to that of αβ T lymphocytes. Consistent with Shamshiev's study [18], we also identified that LA can be presented by different CD1 molecules (CD1b or CD1c). γδ T cells specific to the same antigen but restricted by different CD1 may be recruited in vivo to different anatomical sites depending on the distribution of CD1^+ ^APC [13,19,20].

Our findings demonstrated that recognition of LA by human resting γδ T cells (mainly Vδ2 T cells) depends upon APC, and such recognition is CD1b-/CD1c-dependent and MHC-independent. This conclusion is strongly supported by series of experiments in vitro, especially by antibody blocking assays, proliferative assays using CD1 family molecule-transfected cell lines and pulse-fixation experiments.

CD1 family consists of two groups: group 1 includes CD1a, CD1b and CD1c; group 2 consists of CD1d alone [21]. Several studies [22-24] revealed that the antigen binding sites of CD1 proteins are narrower and deeper than those of MHC molecules and are made up of hydrophobic pockets, which leads to be more adaptive to bind lipid/glycolipid antigens. Our finding raises a question: Why are CD1b and CD1c involved in the presentation of LA for human resting γδ T cells, while CD1a or CD1d is not? Although data from the study of crystallographic structures would explain differences among CD1a, b and d [22-24], further investigation on the function-structure relation is needed to explore the differences among CD1 molecules. Although the crystal structure of CD1c is not clear, we found that the antigen-binding structure between α 1 and α 2 helices of CD1c was more similar to CD1b than to CD1a by computer modeling at the net site http://swissmodel.expasy.org, which is in line with the results obtained from the functional blocking assay. Furthermore, the antibody blocking assay showed that the proliferation of γδ T cells induced by LA-loaded moDC can be almost completely blocked using antibodies against LA and TCRγδ. The result suggests that the recognition of LA by γδ T cell is in a TCRγδ-dependent manner and the epitope of LA recognized by TCRγδ is intact after processed in APC, which was also confirmed by pulse-fixation assay.

Several lines of evidence suggest that murine γδ T cells recognize LPS/LA through TLR2 or TLR4 [25-27]. However, we found that only activated γδ T cells could express both TLR2 and TLR4. It is most important that only TLR4 is much related to LA-loaded moDC-induced proliferation of activated γδ T cells. It seems that activated γδ T cells can recognize LA via TLR4 to magnify their response to LA, which is in agreement with the following report [28]: TLR binding lipid antigens were restricted by the spatial shape of the fatty strain of LA. LA from *Escherichia coli *that adopts a conical shape induces immune response through TLR4, whereas more cylindrical LA from Porphyromonas gingivalis and so on, induces immune response through TLR2.

**Figure 9:**
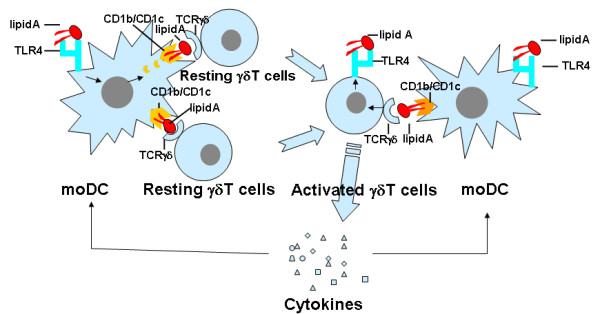
**Schema for the mechanism of interaction of human γδ T cells with Lipid A**.

Because LA is a major component of LPS, γδ T cells, especially Vδ2 T cells in peripheral blood might play an important role in the control of infection induced by Gram-bacteria. Stimulation of γδ T cells with LA results in extensive proliferation and production of abundant Th1 cytokines, especially IFN-γ and TNF-α in vitro, suggesting that LA-activated γδ T cells may play a regulative role for elimination of Gram-bacteria. Based on our study results, we may draw a model (Fig. 9) for the mechanism of interaction of human γδ T cells with Lipid A: in early stage of invasion of Gram-bacteria into epithelium, human resting γδ T cells could receive a signal from APC (such as DC) that is induced by LA associated with CD1b and CD1c and are activated to produce cytokines such as IFN-γ and TNF-α, which then locally strengthens the activity of immune effector cells, such as macrophage. Activated γδ T cells express TLR4, which increase the opportunity of γδ T cells to recognize LA-bearing bacteria and provide additional signal for cell activation. Again, there are many investigations reported that in the presence of LPS and CD1-restricted γδ T cells, immature DCs matured and produced bioactive heterodimeric interleukin-12p70. CD1-restricted γδ T cell recognition of immature DCs provides human immune system with the capacity to rapidly generate a pool of mature DCs early during microbial invasion, which mediate the adaptive response and effectively clear the invading microbes [29,30]. Thus, interaction between γδ T cells and immature DCs should certainly be a benefit for both sides. However, a bridge between them should be (at least include) LA-associated CD1b or CD1c.

## Conclusion

In summary, we found a novel mechanism that human γδ T cells recognize LA in a CD1b- or CD1c-restricted manner in first response against Gram-bacteria, while the interaction between TLR4 on γδ T cells and LA might strengthen the subsequent response of γδ T cells.

## Methods

### Antigen and cytokines

LA (Sigma-Aldrich and Alex) was dissolved at a concentration of 2 mg/ml in 0.1% (v/v) triethylamine solution. The solution was diluted with pyrogen-free PBS or culture medium before use. Isopentenyl pyrophosphate (IPP) is also purchased from Sigma Aldrich and used as positive control. Recombinant human IL-2 (Proleukin), GM-CSF (Berlix) and human IL-4 (Berlix) were used in the study.

### Cells preparation and reagents

PBMCs were obtained by centrifugation of heparinized peripheral blood from different healthy adult donors over Ficoll-Hypaque gradients (Pharmacia). We had received the local Ethical Committee approval and the informed consent of all participating subjects for this investigation. For preparing moDC, fresh PBMCs (5 × 10^6 ^cells/ml) were cultured in 6-well round-bottom microtiter plates (Nunc) for 2 h at 37°C in a humidified atmosphere (5% CO_2_). Then the adherent and nonadherent cells were collected respectively. After washed with RPMI 1640 medium five times, the adherent cells were cultured for 6-8 days on flat-bottom 6-well plates in RPMI 1640 (Gibco) supplemented with 10% FCS (Biowittaker), L-glutamine (Gibco; 2 mmol/L), 1% penicillin-streptomycin Seromed), GM-CSF (50 ng/ml) and IL-4 (25 ng/ml). The moDC were analyzied by flow cytometry and confirmed by light microscope and electron microscope (data not shown). The purity of moDC was > 90%. The percentages of freshly adherent monocytes cells expressing CD14, CD80 and CD86 were 93 ± 2%, 8 ± 1% and 26 ± 5%, respectively. After 7 days of culture in RPMI medium supplemented with human GM-CSF and IL-4, the moDCs became the major population in adherent cells with high percentages of CD80^+ ^(74 ± 4%) and CD86^+ ^(93 ± 3%) cells. Possible contaminating cells were CD14^+ ^monocytes and macrophages, but the percentage was quite low (< 5%). All moDC were irradiated (4,000 rads).

γδ T cell-enriched population was prepared by deleting TCR αβ^+ ^and CD4^+ ^T cells from nonadherent PBMC using CELLection Dynabeads (Dynal Biotech), while purified resting γδ T cells for proliferation assay were prepared by positive selection using anti-γδ-magnetic beads (Miltenyi Biotec), according to the manufacturer's instructions. Cell viability was confirmed by trypan blue exclusion and forward/side-scatter gating. CD1a-, CD1b-, CD1c or CD1d-transfected C1R lymphoblastoma (CD1a-, CD1b-, CD1c- or CD1d-C1R) cell lines were kindly provided by Dr. Peter Cresswell, Yale University, Connecticut, USA and used for the identification of the responses of human γδ T cells to different CD1 family members. These cells were also irradiated (4,000 rads) before use.

### Cytofluorometric analysis

To confirm the quality of moDC, cells were stained by FITC-conjugated anti-CD80, -CD86, -CD1a, CD1b, CD1c, CD1d or -CD14 antibodies (all from Coulter-Immunotech) before and after culture with IL-4 and GM-CSF for 6-8 days. To detect whether there are cross-blocking effects between anti-CD1b and anti-CD1c mAbs, FITC-conjugated anti-CD1b/CD1c mAb was used to stain moDC after blockade by anti-CD1c/CD1b mAb for 2 h (all from Ancell). For evaluation of LA induced-γδ T cell activation,γδ T cell-enriched PBMCs stimulated by LA with or without autologous irradiated moDCs were stained with FITC-conjugated antibody against TCRγδ (Becton Dickinson). To determine the γδ T cell proliferation induced by LA-loaded moDC before and after the antibody blockade, CFSE (2 μM) and phycoerythrin (PE) -conjugated mouse-anti-pan TCRγδ mAb were used. To identify whether freshly isolated/LA-activited γδ T cells express TLR, FITC-conjugated TCRγδ mAb, PE - conjugated anti-pan TLR2 or TLR4 mAb (Becton Dickinson) were used. All the above fluorescence-labeled cells were fixed in PBS with 2% paraformaldehyde and examined using a FACSCalibur flow cytometer (BD Biosciences). Dead cells were excluded on the basis of forward and side light scatter. Data were analyzed using CellQuest or FlowJo software. In all experiments, corresponding isotype-matched mAbs were used as controls.

### ^3^H-TdR incorporation, antibody-blocking and cytokine-detecting assays

Purified γδ T cells (purity 1 × 10^5 ^cells/well) were plated in triplicates in 96-well flat-bottom plates with or without 4000 rads-irradiated moDCs, CD1a-C1R, CD1b-C1R, CD1c-C1R and CD1d-C1R cells (1 × 10^4^/well) as APCs, in the absence or in the presence of LA of 10 ng/ml. To identify which antigen-presenting molecules are involved in LA induced activation of γδ T cell, purified mAbs (25 μg/ml) against MHC I, MHC II, CD1a, CD1b, CD1c or CD1d (BD Biosciences) were preincubated with these APCs. Anti-TCRγδ mAb was also used as a control. For identification of LA antigenicity, polyclonal antibody (pAb) against LA was preincubated with APCs that had been pulsed with LA for 2 h and then washed extensively. In an additional experiment, for the identification of which TLR on LA-activated γδ T cells is involved in the response to LA and the relationship of CD1b/CD1c with TLR2/TLR4, LA-activated γδ T cells were preincubated with mAbs against TLR2, TLR4, or TLR2 plus TLR4 (BD Biosciences) after resting, respectively. Isotype-matched mAbs were used as controls. To determine if LA need processing by moDCs, moDCs were placed in 96-well plates (1 × 10^4^cells/well) and pulsed with LA. After 4 h, cells were washed with RPMI and incubated with 1% paraformaldehyde for 15 min at room temperature. Plates were washed twice with RPMI before addition of 0.2 M lysine. After 20 min, lysine was discarded and plates were washed four times with RPMI. Alternatively, nonpulsed moDCs were fixed and antigen pulse was performed for 4 h. After pulse were carried out, moDCs were washed extensively and 1 × 10^5 ^purified γδ T cells were added to the wells. Cultures were incubated at 37°C in a humidified atmosphere of 5% CO_2 _in air for 48 h, pulsed with 1 μCi of ^3^H-TdR during the last 8 h before harvest using a Tomtec harvester (Hamden CT). The filter paper was counted on a Betaplate scintillatin counter (Wallac). To determine cytokine production, before pulsing with ^3^H-TdR, 50 μl of supernatant in proliferation assay or functional blocking assay was collected from each well. The production of IL-2, IL-4, IL-5, IL-10, IFN-γ and TNF-α was quantified with the CBA Kit according to the manufacturer's instructions (Becton Dickinson). Samples were detected on a flow cytometer (BD FACSCalibur™ flow cytometers) and analyzed through BD CellQuest™ Software.

### TCR δ1^+ ^and TCR δ2^+ ^subset of LA-reactive γδ T cells

To measure the TCRδ_1_^+ ^and TCRδ_2_^+ ^constitution of LA-reactive γδ T cells and their intracellular cytokine production, γδ T cell-enriched PBMCs were co-cultured with LA-pulsed moDCs for 2 weeks. Subsequently, cells were collected and incubated at 3 × 10^6 ^cells/tube at room temperature with anti-CD3 antibody and IL-2 for 6 h and Monensin was added for the final 3 h. The cells were then stained with FITC-conjugated TCRδ_1_^+ ^or TCRδ_2_^+ ^antibody and fixed in 2% paraformaldehyde. Before adding PE-conjugated anti-IFN-γ mAb (BD PharMingen) or control PE-conjugated mouse IgG1, the cells were permeabilized with 0.5% saponin at RT for 1 h. A gate was set on TCRδ_1_^+ ^or TCRδ_2_^+ ^population, and the percentage of IFN-γ positive cells were examined using a FACSCalibur flow cytometer.

### Statistical analysis

Statistical analysis was determined by Student's *t *test and a *P *of < 0.05 was considered significant.

## Competing interests

The authors declare that they have no competing interests.

## Authors' contributions

YC carried out the experiments and drafted the manuscript. LK participated in the preparation of PBMC and performed the statistical analysis. WH and LC conceived of the study and parcipated in its design and coordination and helped to draft the manuscript. All authors approved the final version.

## Reviewers' comments

### Reviewer's report 1

Dr. Hao Shen

Department of Microbiology, U PENN School of Medicine, USA.

*In this study, Cui et al. show that lipid A component of LPS is an antigen presented by CD1 on monocyte-derived dendritic cells (DC) to gamma delta (γδ) T-cells. The specificity of human blood γδ T cells is still largely undefined, except for isoprenyl pyrophosphates (IPP) or related, microbial-derived HMB PP, and related artificial compounds. The study by Cui et al. adds lipid A as an alternative ligand for human blood γδ T cells and, therefore, is novel and potentially of substantial interest to γδ T cell research. The experiments supporting their conclusion include γδ T cell proliferation and cytokine secretion in response to lipid A. Furthermore, highly convincing data are presented that show the involvement of CD1b and CD1c in this process. Recognition of IPP or related, microbial-derived HMB PP is in line with the tremendous expansion of this T cell subset in the setting of many microbial infections. Similarly, recognition of lipid A as shown in this study provides further evidence for a role of γδ T cells in microbial infection. These results further our understanding the recognition properties of these important human T cells and their biological function in the context of a role of γδ T cells in innate immune responses*.

Two major issues need to be addressed:

#### Specific point 1

*The purity of the lipid A material is of utmost importance for this studies. Any containmination of TLR ligands would compound the interpretation of the results*.

*Author's Response*

We totally agree with the referee that the purity of lipid A is very important, because some phosphoantigens such as HM-PP (or related compounds) that may mixed with the lipid A preparation can induce very strong expansion of γδ T even at very low cencentrations (< nM) and we have repeated the proliferation experiments five times, including three experiments using purified lipid A from Sigma Aldrich and two experiments using *the *"TLR grade" > 99.9% pure lipid A available from Alexis. The results obtained from all of the experiments are consistent, Lipid A induced significant expansion of γδ T cells in the presence of autologous moDC.

#### Specific point 2

*Since the subset of γδ T cells (i.e., Vd2) activated by lipid A in this study also recognize the phosphoantigen, it would have been very informative to include a standard phosphoantigen such as IPP or BrHPP for comparison. One would expect strong expansion of γδ T cells in response to phosphoantigens in the absence of added DCs (in apparent contrast to lipid A)*.

*Author's Response*

Yes, it is really the case. As we expected, IPP indeed induced significantly expansion of purified γδ T cells when we repeated the ^3^H-TdR incorporation assay using IPP as positive control.

### Reviewer's report 2

Dr. Youwen He (nominated by Dr. Laurence C Eisenlohr, Thomas Jefferson University),

Department of Immunology, Duke University Medical Center, USA.

*The manuscript by Cui et al has investigated the recognition mechanism of lipid A, an important microbial component, by human gamma delta T cells. The authors first demonstrated that human γδ T cells can be induced to undergo strong proliferation by lipid A pulsed antigen presenting cells. The proliferation of human γδ T cells can be blocked by antibodies against CD1b and CD1c but not CD1a or CD1d. To further investigate the specificity of the interaction, the authors used CD1b or CD1c transfected C1R lymphoblastoma cells as antigen presenting cells. The antibody blocking experiments again demonstrated that CD1b and CD1c play critical roles in presenting lipid A to human γδ T cells to induce cell proliferation. Interestingly, lipid A stimulated human γδ T cells produce a Th2 cytokine profile*.

*The experiments are well designed and the results are solid. The findings presented in this manuscript are significant in that it has identified a novel mechanism for human γδ T cells in recognition of microbial component. I recommend acceptance of this manuscript*.

#### Specific point 1

*The antibody blocking experiments in *Figure 2* showed that anti-CD1b or CD1c, but not CD1a or CD1d, readily blocked lipid A induced proliferation. Have the authors tested to use a combination of CD1a and CD1d for the blocking assay?*

*Author's Response*

We ever examined the combined blocking function of anti-CD1a and CD1d mAbs through ^3^H-TdR incorporation assay in our pretest, but just as what they act alone, the proliferation of γδ T cells in this group is not significantly different from the group without any mAb (*p > 0.05*).

#### Specific point 2

*In *Figure 4B, *the panel labels (A and B) are not clear. Please change the labels*.

*Author's Response*

Changed.

#### Specific point 3

*What are the statistic values among the different groups in *Figure 5B?

*Author's Response*

We have made a statistic analysis and corresponding revision in manuscript and Fig. 8 according to the statistic results, which showed that when compared with γδ T cells in the group without any mAb, the proliferation of γδ T cells respectively in the groups with single or both of anti-CD1b and CD1c mAbs, the group with mAbs against CD1b, CD1c, TLR4 and TLR2, or the group with mAb against TLR4 exhibited significant difference (P < 0.05). However, The group with mAb against TLR2 exhibited no significant difference (P > 0.05).

### Reviewer's report 3

Dr. Michael Lenardo and Dr. Pushpa Pandiyan,

Molecular Development Section, Laboratory of Immunology, NIH, USA.

*The authors have put forward an interesting concept on host immune defense by demonstrating γδ T cell dependent response to lipid antigen bearing bacteria. They deserve credit for presenting a concise report of their findings with clearly presented data. They have demonstrated that recognition of LA by human resting γδ T cells (mainly Vδ2 T cells) depends upon APC, and this recognition is CD1b-/CD1c-dependent and MHC-independent*.

*There are two important questions that need to be addressed before the manuscript can be published in "Biology Direct" journal*.

*Because the whole manuscript is based on γδ T cells response to LA, the purity of the starting population of γδ T cells and LA is very crucial*.

#### Specific point 1

*The authors have not shown the purity of starting population. By 1 week after stimulation, they are only 23% γδ positive *(Fig. 1A). *This data raises an important concern of whether the response is an indirect effect on the non γδ T cells that may be expressing TLRs in their culture. The authors should perform at least one experiment showing CFSE dilution of TCR γδ positive cells using highly purified γδ T cells as starting population*.

*Author's Response*

We completely agree with the comments and have made corresponding description in the text now. As shown in Fig 1, TCR γδ expressing cell respectively occupied 4% and 21% in freshly isolated PBMC and γδ T cell-enriched PBMC obtained from depleting TCRαβ^+ ^and CD4^+ ^T cells in the representative donor. We used these γδ T cell-enriched PBMC as starting population in Flow Cytometry analysis and CFSE dilution assay (Fig. 2) to detect the proliferation of human resting γδ T cell induced by LA.

Based on the advice of Dr. Michael Lenardo and Dr. Pushpa Pandiyan, we repeated the CFSE dilution assay using highly purified γδ T cells as starting population (99% purified), although it needs much more fresh blood. The results were consistent with what we previously obtained, indicating that it was the LA-loaded moDCs that induce significant proliferation of human γδ T cells.

#### Specific point 2

*LPS contamination in LA can be addressed easily. For example, the authors should demonstrate how B cells respond to the LA preparations they used*.

*Author's Response*

We had confirmed the purity of Lipid A preparation we used by HPLC and there was no evidence showing molecules like LPS in it. As for the mechanism of B lymphocytes interaction with lipid A, we might dwell on it in a future study project.

#### Minor point

*On Page 11, the word ****"Invasing"*** *should be changed ****to "invading"*** *in the sentence, "....DCs early during microbial invasion, which mediate the adaptive response and effectively.... clear the invasing microbes"*

*Response*

Changed.
